# Effective inactivation of *Bacillus atrophaeus* spores and *Escherichia coli* on disposable face masks using ultraviolet laser irradiation

**DOI:** 10.1186/s40543-022-00332-7

**Published:** 2022-06-27

**Authors:** My-Chi Thi Nguyen, Huu-Quang Nguyen, Hanbyeol Jang, Sojung Noh, Youngku Sohn, Kiju Yee, Heesoo Jung, Jeongkwon Kim

**Affiliations:** 1 Department of Chemistry, Chungnam National University, 99 Daehak-Ro, Yuseong-Gu, Daejeon, 34134 Republic of Korea; 2 Department of Chemical Engineering and Applied Chemistry, Chungnam National University, Daejeon, 34134 Republic of Korea; 3 Department of Physics and Institute of Quantum Systems, Chungnam National University, Daejeon, 34134 Republic of Korea; 4 Chem-Bio Technology Center, Agency for Defense Development (ADD), Yuseong P.O. Box 35, Daejeon, 34186 Republic of Korea; 5 Graduate School of New Drug Discovery and Development, Chungnam National University, Daejeon, 34134 Republic of Korea

**Keywords:** Ultraviolet, Laser irradiation, Sterilization, Face masks, *Bacillus* spores, *Escherichia coli*

## Abstract

Due to the widespread emergence of COVID-19, face masks have become a common tool for reducing transmission risk between people, increasing the need for sterilization methods against mask-contaminated microorganisms. In this study, we measured the efficacy of ultraviolet (UV) laser irradiation (266 nm) as a sterilization technique against *Bacillus atrophaeus* spores and *Escherichia coli* on three different types of face mask. The UV laser source demonstrated high penetration of inner mask layers, inactivating microorganisms in a short time while maintaining the particle filtration efficiency of the masks. This study demonstrates that UV laser irradiation is an efficient sterilization method for removing pathogens from face masks.

## Introduction

Face masks are commonly used to prevent inhalation of environmental air pollutions such as yellow dust and fine particles. During the COVID-19 pandemic, face masks and facepiece respirators have been an important aspect of reducing the risk for human-to-human transmission of COVID-19 through bioaerosols and droplets. However, due to a shortage of personal protective equipment, the reuse of face masks is often required (Celina et al. 2020; Cramer et al. 2021), highlighting the importance of sterilization methods to reduce microorganisms on face masks prior to rewear. A proper sterilization method would also help to sterilize contaminated respirators before disposal, keeping them out of the environment (Hossain et al. 2015; Zhao et al. 2021).

A growing number of sterilization methods have been introduced to eliminate microorganisms such as viruses, bacterial cells, and bacterial spores on face masks and respirators (Rodriguez-Martinez et al. 2020). Dry heat is effective for inactivating viruses and bacteria after 2 h exposure but causes a sharp reduction in particle filtration efficiency with temperatures higher than 100 °C (Oh et al. 2020; Pascoe et al. 2020). Chemical-based agents such as hypochlorite, hydrogen peroxide, ethanol, isopropanol, and detergents also decrease filtration efficiency, and lingering chemical residues can generate unpleasant odors and damage the skin (Derraik et al. 2020; Jung et al. 2020; McEvoy et al. 2019; Viscusi et al. 2009). While gamma irradiation is widely used to sterilize medical devices, it also reduces the filtration performance of respiratory devices (DeAngelis et al. 2021). Exposure to ultraviolet C (UV-C, 200–280 nm) light is one of the most commonly used sterilization methods (Jang et al. 2022) because it is effective against bacterial cells and spores with little to no effect on filtration efficiency in respiratory devices.(Lindsley et al. 2015; Nguyen et al. 2021; Paul et al. 2020). The most challenging aspect of the use of UV radiation is that microorganism sterilization can vary between non-porous and porous surfaces due to a shadowing effect (Banerjee et al. 2021; Kayani et al. 2021). There is concern that UV radiation will have reduced sterilization efficiency on inner layers of masks which are not directly exposed to the UV source.

Pathogens from both spore-forming and non-spore-forming bacteria pose potential threats to public health (Post 2019). *Bacillus* are spore-forming, Gram-positive species whose spores are metabolically dormant and greatly resistant to disinfectants. *Bacillus* strains are associated with food poisoning (*Bacillus cereus*) (Stenfors Arnesen et al. 2008), insect pathogens (*Bacillus thuringiensis* and *Bacillus sphaericus*) (Aronson et al. 1986), and bioterrorism agents (*Bacillus anthracis*) (Spotts Whitney et al. 2003). *Escherichia coli* (*E. coli*) is a non-spore-forming, Gram-negative bacterium commonly found in the intestines of people and other animals. *E*. *coli* O157:H7 leads to severe stomach cramps, bloody diarrhea, vomiting, and acute kidney failure (Armstrong et al. 1996; Doyle 1991). Multidrug-resistant strains of *E*. *coli* have also been reported to interfere with the treatment of bloodstream (Paramita et al. 2020) and urinary tract infections (Manges et al. 2001), and when a host is immunosuppressed or when gastrointestinal barriers are invaded, even non-pathogenic *E*. *coli* can cause infections (Kaper et al. 2004).

This study investigated the sterilization of *Bacillus atrophaeus* spores and *E*. *coli* on face masks using a neodymium-doped yttrium orthovanadate (Nd:YVO_4_) UV laser (266 nm, 1 W) with different numbers of scans. The impact of laser treatment was quantitatively examined according to the viability of microorganisms and the particle filtration efficiency of the masks.

## Materials and methods

### Bacterial strains

*Bacillus atrophaeus* spores (PTG706) and *E*. *coli* (PTG708) strains were purchased from Protigen (Protigen Corp., Jeonju, Jeollabuk-do, Korea). Both standard colony-forming units were ~ 10^8^ CFU/mL. All strains were stored at 4 °C.

### Disposable face masks

**Fig. 1:**
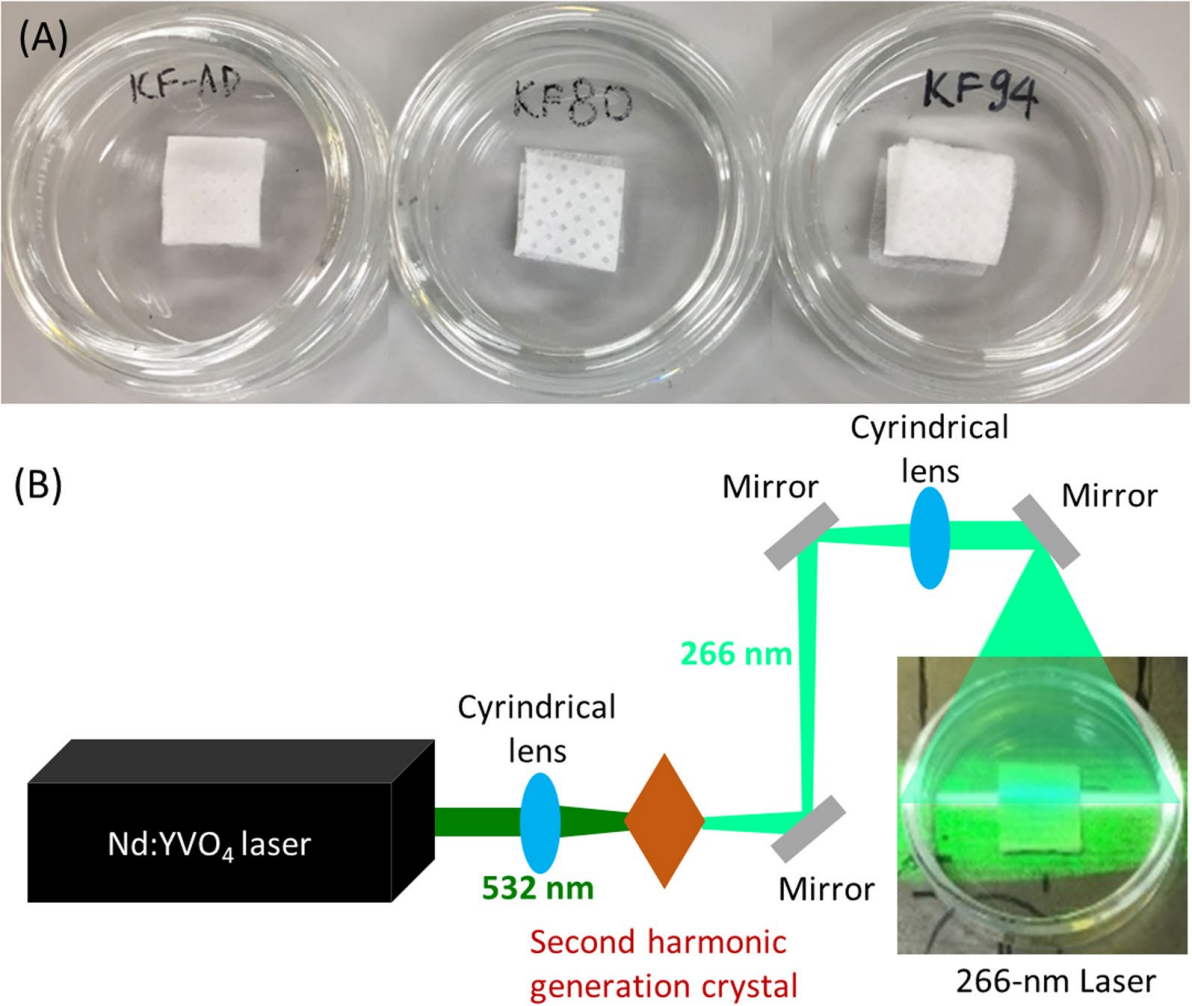
**A** KF-AD, KF80, and KF94 face masks (1 × 1 cm), **B** schematic of laser irradiation of a face mask

Three brands of disposable face mask were purchased and used for the investigation: an anti-droplet mask (Korean Filter [KF]-AD) and two safety face masks (KF80 and KF94) certified by the Ministry of Food and Drug Safety (MFDS, formerly known as the Korea Food & Drug Administration or KFDA). The KF-AD is a type of light mask for easy breathing and that prevents droplet inhalation. The KF80 and KF94 have filtration efficiencies of > 80% for 0.6 µm particles and > 94% for 0.4 µm fine particles (MFDS 2020). The KF94 is equivalent to the N95 respirator mask approved by the United States National Institute for Occupational Safety and Health, and to FFP2 masks approved by the European standard (Park 2020). Each mask layer was optically analyzed using an optical microscope (Leica EZ4, Leica Microsystems, Wetzlar, Germany). These masks were cut into small pieces of 1 × 1 cm (Fig. 1A) and autoclaved prior to bacterial loading.

### Optimization of adsorption solvent and absorption time

Adsorption solvent was determined by diluting stock solutions of *Bacillus atrophaeus* spores and *E*. *coli* (10^8^ CFU/mL) in one of three different solvents to make 10^6^ CFU/mL solution: deionized water, 40% ethanol, and phosphate-buffered saline (PBS). Then, 20 µL each solution was loaded onto the mask pieces. Various adsorption durations were also examined to determine the optimized adsorption time. *Bacillus atrophaeus* spores and *E*. *coli* (10^6^ CFU/mL, 20 µL) solutions were individually loaded onto the surface of the KF94 masks and allowed to dry in a biological safety cabinet (Labconco 4FT, Labconco Corp., Kansas, MO, USA) for up to 24 h.

### Laser irradiation

Contaminated masks were treated using a 266 nm UV laser generated by second-harmonic generation of a 532 nm Nd:YVO4 laser (Lee et al. 2021). The laser beam was generated at an energy of 1.0 ± 0.3 W, a pulse width of 50 ns, a repetition rate of 120 kHz, diameter of 7 cm, speed of 5.0 ± 0.5 mm/s, and scan distance of 1 cm from a nanosecond pulsed laser AVIA 532–45 (Coherent Inc., Santa Clara​, CA, USA). The laser beam was able to move back and forth. A picture showing the laser irradiation process is displayed in Fig. 1B. Samples were irradiated by 1, 2, and 12 UV laser scans for 5, 10, and 60 s, respectively. Untreated spores on masks were prepared as controls. Each experiment was conducted in triplicate.

### Measurement of bacterial survival

*Bacillus atrophaeus* and *E*. *coli* samples were released from the masks after a specified adsorption time or laser irradiation scan by mildly scratching the mask surface with pipette tips and repeated pipetting of 300 µL PBS with 0.02% Tween 20. Then, a portion (25 µL) of the detached solution was evenly smeared onto a nutrient-rich LB agar plate and incubated at 37 °C for 16–24 h. Viable colonies were counted in ImageJ, and CFU values were calculated. If the number of viable colonies was too high to count, dilution prior to incubation was performed. In the case of no viable colonies, CFU values were assigned as 1 (Yadav et al. 2004). To clearly display the change in the number of colonies, survival fraction was defined as below (Cortesao et al. 2020).

Survival fraction = *N*/*N*_0_.

*N*: mean CFU recovered from irradiated sample.

*N*_0_: mean CFU recovered from untreated sample.

Log_10_(survival fraction) is equal to –log_10_(*N*_0_/*N*), where log_10_(*N*_0_/*N*) is commonly known as log_10_(reduction) (Wood et al. 2019).

### Particle filtration efficiency

A whole mask, approximately 210 × 80 mm, was irradiated with varying numbers of UV laser scans. Particle filtration efficiency was measured using a filter tester (DL-360F, Daelim Starlet Co., Ltd., Siheung-si, Republic of Korea) equipped with a sodium chloride (NaCl) generator for KF-AD and KF80 masks and a paraffin oil aerosol generator for KF94 masks. Average particle sizes for NaCl and paraffin oil aerosols are 0.6 μm and 0.4 μm, respectively. Aerosol flow rate was 95 L/min with a concentration of 8 ± 4 mg/m^3^ (NaCl) or 20 ± 5 mg/m^3^ (paraffin oil) passed over the masks. After 3 min, particle concentrations were recorded in 30 ± 3 s intervals between measurements. The particle filtration efficiency was calculated using the ratio of the particle concentration kept by masks (*C*_1_-*C*_2_) and initial particle amount (*C*_1_) as shown below.

$$P(\% ) = \frac{{C_{1} {-}C_{2} }}{{C_{1} }} \times 100$$

*P*: Particle filtration efficiency.

*C*_1_: Pre-passage concentration of NaCl or paraffin oil.

*C*_2_: Post-passage concentration of NaCl or paraffin oil.

## Results and discussion

### Structure of the face mask layers

**Fig. 2:**
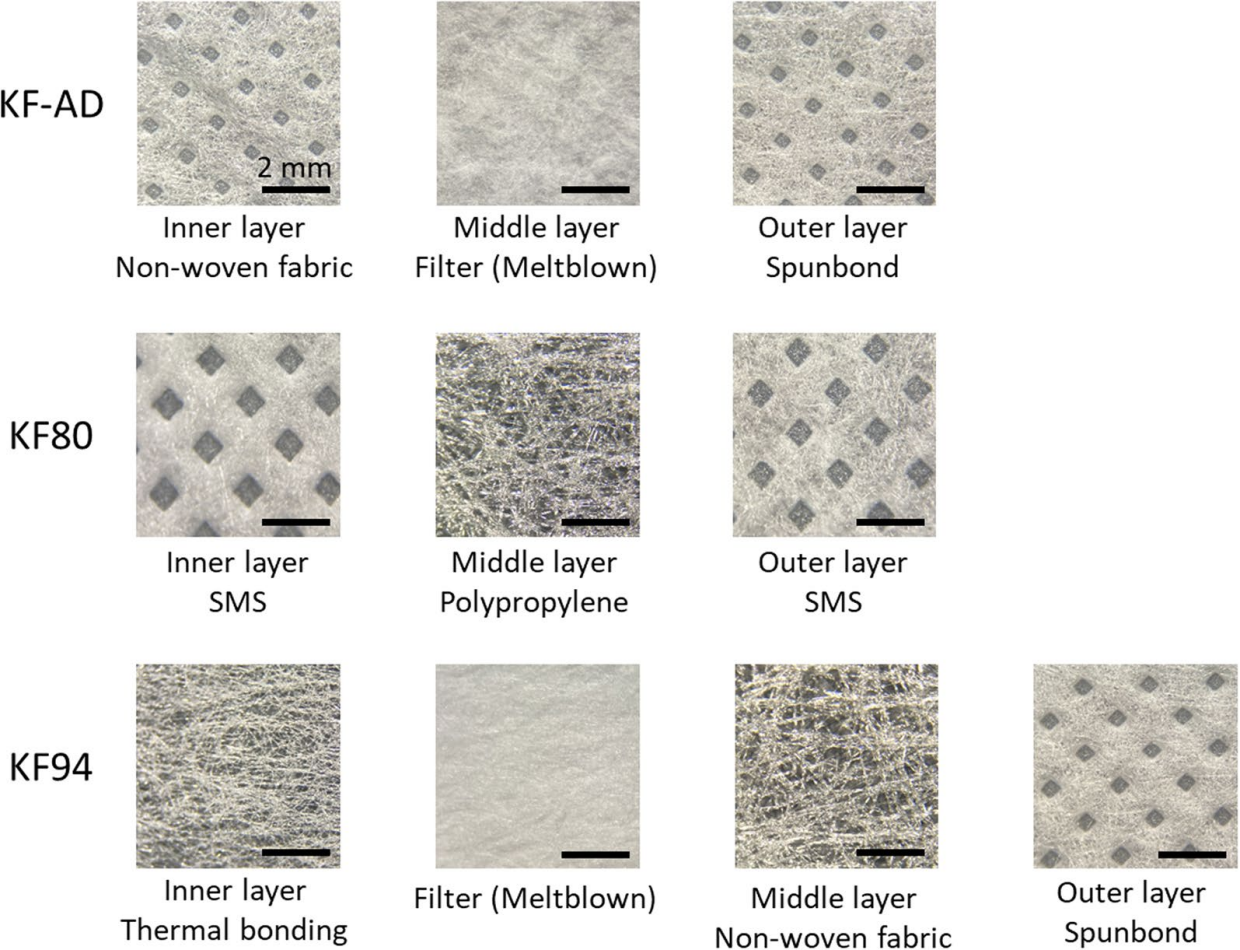
Structure of mask layers of KF-AD, KF80, and KF94 visualized through an optical microscope

The structure of each mask type was examined using an optical microscope as shown in Fig. 2. KF-AD masks are composed of a non-woven fabric inner layer, a meltblown filter, and a spunbond outer layer. KF80 masks are composed of a polypropylene middle layer between two spunbond–meltblown–spunbond (SMS) layers. KF94 is a 4-ply mask with a spunbond outer layer, a meltblown filter, a thermal bonding middle layer, and an inner layer of non-woven fabric.

### Optimization of adsorption conditions

#### Selection of adsorption solvent

The face masks are structurally composed of several stacked layers (Jung et al. 2020). Due to the hydrophobic nature of the mask layers, samples diluted in deionized water had difficulty binding to the mask surface (Li et al. 2006). Thus, the use of an alternative solvent was necessary.

To reduce the amount of water in the adsorption solvent, an aqueous ethanol solution was considered an alternative adsorption solvent. Although *Bacillus* spores are highly resistant to ethanol, the relative survival of *Bacillus* spores was significantly decreased after diluting them in solutions containing more than 50% ethanol (Lin et al. 2018). Therefore, 40% ethanol was used as a adsorption solvent for *Bacillus atrophaeus* spores. Use of this solvent caused gradual absorption of spores into the mask layers. By contrast, even low concentrations of ethanol have adverse effects on the viability of *E*. *coli* particles (Elzain et al. 2019; Horinouchi et al. 2010). Thus, PBS was used to dilute *E*. *coli*. In contrast to deionized water, PBS was more easily adsorbed into the layers.

#### Selection of adsorption time

*Bacillus* spores are resistant to stress and have a long survival time on surfaces (Brosseau et al. 1997; Setlow 2006), while *E*. *coli* survival drastically decreases after 8 h on respirators (Lin et al. 2017). Water loss from long-term storage may damage the cell membrane and lead to protein misfolding and detrimental effects on nucleic acids and lipids (Billi et al. 2002), thus interfering with the viability of *E*. *coli* following culture. To determine the optimum adsorption duration for *Bacillus* spores and *E*. *coli*, the bacteria were detached and cultured to obtain the survival fraction. In the medium of LB agar, *Bacillus* spores after incubation form circular, light-orange colonies (Gibbons et al. 2011). Meanwhile, *E*. *coli* colonies are off-white with a shiny texture (Son et al. 2012).

**Fig. 3:**
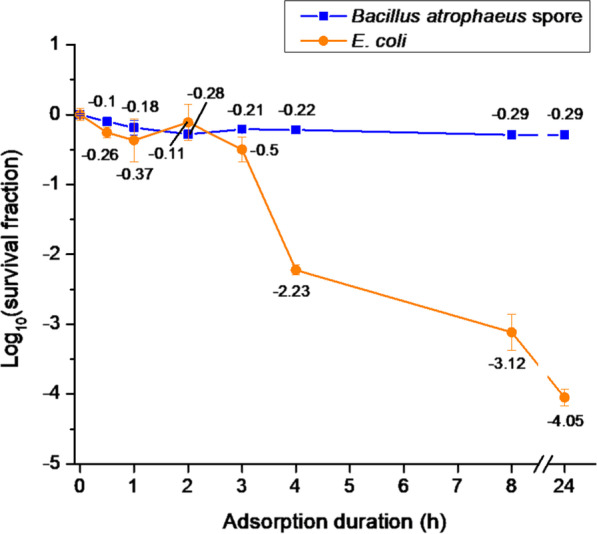
Effect of adsorption time on KF94 masks on viability of *B. atrophaeus* and *E*. *coli*

There were no significant differences in the viability of *Bacillus atrophaeus* spores (10^6^ CFU/mL, 20 µL) loaded on the KF94 respirator masks up to 24 h (Fig. 3). The log_10_(survival fraction) was − 0.10 after 30 min and around − 0.28 from 2 to 24 h. *E*. *coli* (10^6^ CFU/mL, 20 µL) showed a significant reduction in survival fraction with increasing adsorption duration time where the log_10_(survival fraction) was − 0.50 after 3 h adsorption and significantly decreased to − 2.23 after 4 h adsorption. The values further decreased to − 3.12 and − 4.05 after 8 and 24 h, respectively. Although the adsorption duration up to 24 h on face masks did not result in any adverse effect on viability of *Bacillus* spores, a notable reduction of *E*. *coli* was observed after 3 h. Therefore, we investigated the effect of UV laser irradiation in masks treated with *Bacillus* spores after drying overnight (16 h), and masks treated with *E*. *coli* after 2 h adsorption.

### Measurement of bacterial survival

**Fig. 4:**
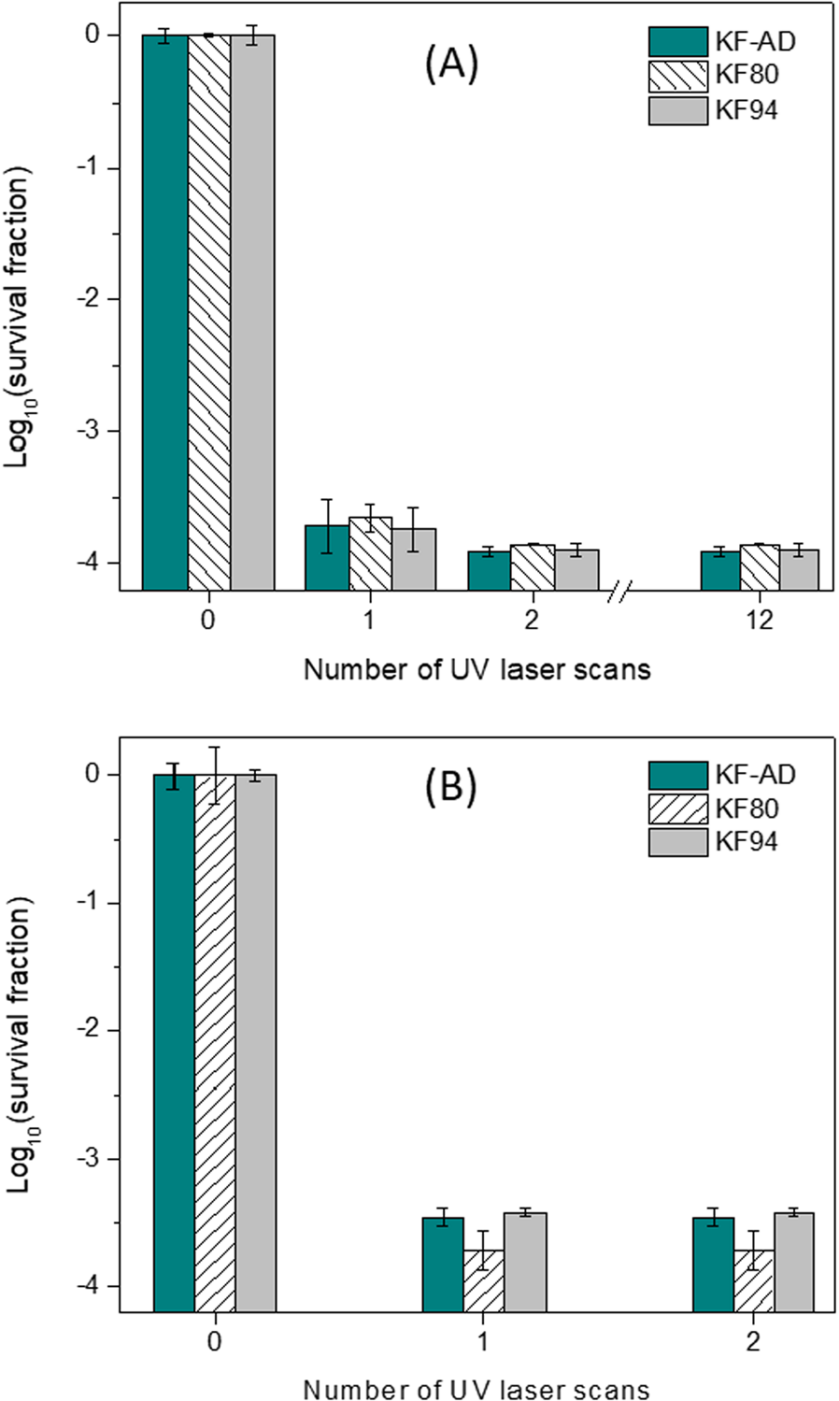
LR values of **A** *B. atrophaeus* spores irradiated with 1, 2, and 12 UV laser and **B** *E*. *coli* irradiated with 1 and 2 UV laser

To investigate the sterilization effect of UV laser irradiation, bacterial samples were adsorbed on the masks for 16 h (*Bacillus* spores) and 2 h (*E*. *coli*). After exposure to the UV laser, bacterial samples were detached and cultured to determine remaining viability. Figure 4A shows the log_10_(survival fraction) of *Bacillus atrophaeus* spores. Treatment with the UV laser led to a log_10_(survival fraction) of − 3.71, − 3.65, and − 3.74 corresponding to KF-AD, KF80, and KF94 masks after 1 scan. Increasing the number of UV laser scans led to increased inactivation of bacterial particles, with a log_10_(survival fraction) of − 3.91, − 3.85, and − 3.89 for KF-AD, KF80, and KF94 masks, respectively, after 2 and 12 scans. In masks treated with *E*. *coli*, only 1 UV laser scan was needed for complete inactivation of KF-AD, KF80, and KF94 masks with log_10_(survival fraction) of − 3.45, − 3.72, and − 3.42, respectively (Fig. 4B). Because *E*. *coli* is vulnerable to dehydration, it is also more sensitive to UV laser than bacterial spores.

Decreased bacterial viability was similar between the different types of face mask, suggesting that the number of layers and type of material do not impact the effectiveness or penetration of the UV laser.

### Particle filtration efficiency

**Fig. 5:**
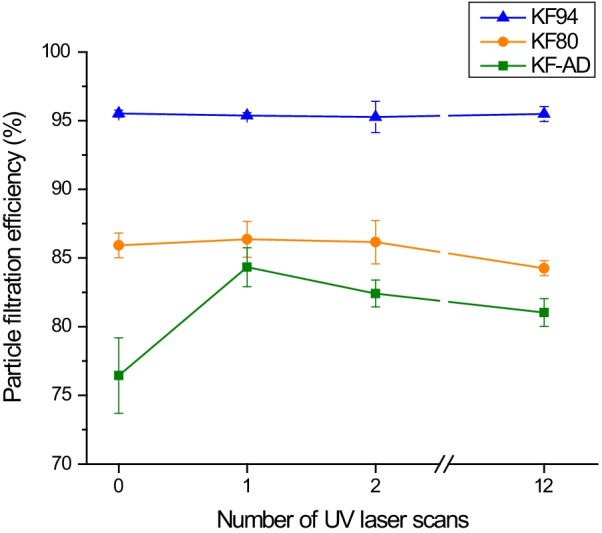
Particle filtration efficiency of KF-AD, KF80, and KF94 masks before and after 1, 2, and 12 UV laser irradiation

To investigate whether UV laser irradiation would decrease the performance of the face masks, we measured the particle filtration efficiency of NaCl and paraffin oil aerosols before and after UV laser irradiation. As demonstrated in Fig. 5, the masks demonstrated a high filtration efficiency (of 76.4%, 85.9%, and 95.5% for KF-AD, KF80, and KF94, respectively). After irradiation, KF94 masks retained a 95% filtration efficiency against paraffin oil filtration. The NaCl filtration efficiency decreased slightly after irradiation for the KF80 and KF-AD masks at 84.3% and 81.0% after 12 laser scans. Therefore, the physical characteristics of the three masks were not significantly affected by UV laser irradiation up to 12 scans.

The deterioration of particle filtration efficiency from sterilization treatments has been a limiting factor in the ability to reuse masks. Although alcohol solutions are effective at removing attached bacteria, they also reduce filtration performance (Kim et al. 2009; Ullah et al. 2020). Washing masks with or without detergent can also destroy filter integrity (Jung et al. 2020; Viscusi et al. 2007). Conversely, while UV irradiation-based treatment does not impact filtration efficiency, there is incomplete sterilization against *E*. *coli* (Jung et al. 2020). There are some limitations of using 266 nm pulsed laser for general purpose due to its high cost, high energy consumption, and trained operator requirement. The current results, however, show that UV laser irradiation could be used as alternative sterilization method for face masks in regard to both *Bacillus* spores and *E*. *coli*. Further research of UV laser irradiation is needed to include face mask components such as straps and metal nose clips.

## Conclusions

Irradiation with a Nd:YVO_4_ laser (266 nm, 1 W) is an effective sterilization method against both *Bacillus atrophaeus* spores and non-spore-forming bacteria of *E*. *coli* contaminating three different kinds of safety face mask (KF-AD, KF80, and KF94). The particle filtration efficiency was successfully preserved after UV laser irradiation regardless of the type of investigated material.

## Data Availability

Not applicable.
